# An integrative comparative study between ultrasound-guided regional anesthesia versus parenteral opioids alone for analgesia in emergency department patients with hip fractures: A systematic review and meta-analysis

**DOI:** 10.1016/j.heliyon.2022.e12413

**Published:** 2022-12-19

**Authors:** Hany A. Zaki, Haris Iftikhar, Nabil Shallik, Amr Elmoheen, Khalid Bashir, Eman E. Shaban, Aftab Mohammad Azad

**Affiliations:** a Emergency Medicine, Hamad General Hospital, P.O. Box 3050, Doha, Qatar; b Anesthesia Department, Hamad Medical Corporation, Doha, Qatar; c College of Medicine, Qatar University, Doha, Qatar; d Weill Cornell Medical College in Qatar, Doha, Qatar; e Cardiology, Al Jufairi Diagnosis and Treatment, Doha, Qatar

**Keywords:** Ultrasound, Regional anesthesia, Opioids, Adverse effects, Emergency, Hip fracture, Systematic review, Meta-analysis

## Abstract

**Background:**

Emergency physicians play a major role in managing patients with hip fractures. The most commonly used pain management option is parenteral opioids. However, parenteral opioids are subjected to several adverse effects. New pain management techniques such as regional anesthesia are used as alternatives to parenteral opioids. Anatomical landmarks were used to administer regional anesthesia; however, ultrasound guidance has shown promising results with regional anesthesia.

**Objective:**

of the Review: The present study compares the efficacy of ultrasound-guided regional anesthesia (USGRA) to parenteral opioids in analgesia of hip fractures patients.

**Methods:**

A literature search for original and relevant articles carried out through six electronic databases, yielded 710 articles which were then assessed using the eligibility criteria resulting in 8 studies eligible for inclusion.

**Results:**

A Meta-analysis of the seven studies showed that ultrasound-guided femoral nerve block was more effective than parenteral opioids in relieving pain. Similarly, meta-analysis of data from two studies shows that US-guided FICB significantly reduced pain scores than parenteral opioids. A subgroup analysis of adverse events showed no significant difference in nausea/vomiting and respiratory complications. However, a subgroup analysis on hypotension showed that the incidence of hypotension was significantly lower in USGRA than parenteral opioids. The present study also revealed that patients in the USGRA group required less frequent rescue analgesia than the patients in the parenteral opioids group.

**Conclusion:**

Results of the present study show that USGRA is superior to parenteral opioids in reducing pain and the need for rescue analgesia in patients with hip fractures.

## 1 Introduction

Hip fractures are common severe injuries in older patients leading to a public health concern. It is estimated that 1 in every 3 women and 1 in every 12 men will develop hip fractures in their lifetime [1]. An earlier study found that people aged 65 and older account for 86% of hip fracture cases [2]. Evidence has also suggested that hip fractures increases the risk of mortality, morbidity, functional impairment, and financial strain [2, 3, 4]. The majority of patients with hip fractures seek care in the emergency room (ER), so emergency physicians are crucial to the management of these patients. However, providing safe and efficient pain management for hip fracture patients in the ER can be challenging.

The most commonly used pain management analgesics are parenteral opioids. These opioids are often associated with adverse reactions, and the risks are significantly higher in older patients [5]. Therefore, to minimize the adverse events, the analgesics are provided in low dosages under patient control resulting to a substantial consumption of healthcare resources. For this reason, alternative pain management techniques have been taken into consideration for older patients with hip fractures. One of the alternative pain management methods used in the emergency department (ED) for patients with hip fractures is regional anesthesia. Unlike parenteral opioids regional anesthesia targets a specific area of the body to alleviate pain, thus resulting in fewer adverse reactions. A Previous study reported that regional anesthesia, such as three-in-one femoral nerve block (FNB) is a promising alternative to opioids in patients with hip fractures [6]. However, it is reported that this method has not gained widespread application because it is considered a “blind” procedure that can be risky [7].

Recently, ultrasound guidance has been used as an alternative to safely conduct regional anesthesia in hip fracture patients presented to the ED. The use of ultrasound-guided regional anesthesia has been associated with several advantages, including the widespread availability of ultrasound in the ED, emergency physicians’ skills and comfortability to use ultrasound, and the ability of the ultrasound-guided procedure to visualize the femoral neurovascular anatomy. It has also been reported that ultrasound-guided regional anesthesia (USGRA) may be superior to other regional anesthesia with regard to the onset of action and the amount of anesthetic required [8, 9].

To the best of our knowledge, systematic reviews comparing USGRA to parenteral opioids alone have not been carried out. Therefore, the primary goal of this systematic review and meta-analysis was to compare the efficacy of USGRA to parenteral opioids alone in reducing pain for patients with hip surgery. This study also compares the adverse events observed in patients receiving either USGRA or parenteral opioids. We hypothesize that USGRA will significantly lower the pain intensity and have significantly fewer adverse events than parenteral opioids.

## 2 Methodology

### 2.1 Literature search

To find all studies that compared UGRA to parenteral opioids alone, an independent search in 6 databases, including PubMed, Cochrane central register of controlled trials, Medline, ScienceDirect, Embase, and Google Scholar was performed. Boolean expressions “AND” and “OR” were combined with specific keywords to form the following search strategy; (“Ultrasound-guided” OR “Ultrasound”) AND (“regional anesthesia” OR “nerve block” OR “peripheral nerve block” OR “fascia iliaca compartment block” OR “femoral nerve block” OR “3-in-1 blocks” OR pericapsular nerve group Page block”) AND (“parenteral opioids” OR “intravenous opioids” OR “intramuscular opioids”) AND (“Hip fracture” OR “Femur head fracture” OR “femoral fractures” OR “Trochanteric fractures” OR “Subtrochanteric fracture” OR “Extracapsular fracture” OR “Intracapsular fracture”). Articles relevant to our study also had their reference lists scrutinized for additional studies.

### 2.2 Eligibility criteria

#### 2.2.1 Inclusion criteria

Once the studies related to our topic were identified, two reviewers used the inclusion and exclusion criteria to determine which studies were eligible for inclusion. The following criteria were used when including studies in the current systematic review;

i. Scientific publications written in English. This specification was developed to prevent the loss of context and meaning when directly translating scientific terms.
ii. Studies conducted on human subjects only.
iii. Studies comparing any ultrasound-guided regional anesthesia to any parenteral opioids.
iv. Studies conducted on patients with hip fractures or femur fractures or set to undergo hip surgery.

#### 2.2.2 Exclusion criteria

The following criteria were used to excluded articles from the current review:

i. Studies including animal subjects
ii. Studies independently evaluating either any USGRA or parenteral opioids on patients with hip fractures.
iii. Studies comparing regional anesthesia to other analgesics on patients with hip fractures
iv. Studies comparing USGRA to parenteral opioids on patients with fractures of the upper extremity or knee injuries
v. Letters to the editor, other systematic reviews, meta-analyses, cadaveric studies, and case reports were also excluded.

### 2.3 Quality assessment

**Figure 1:**
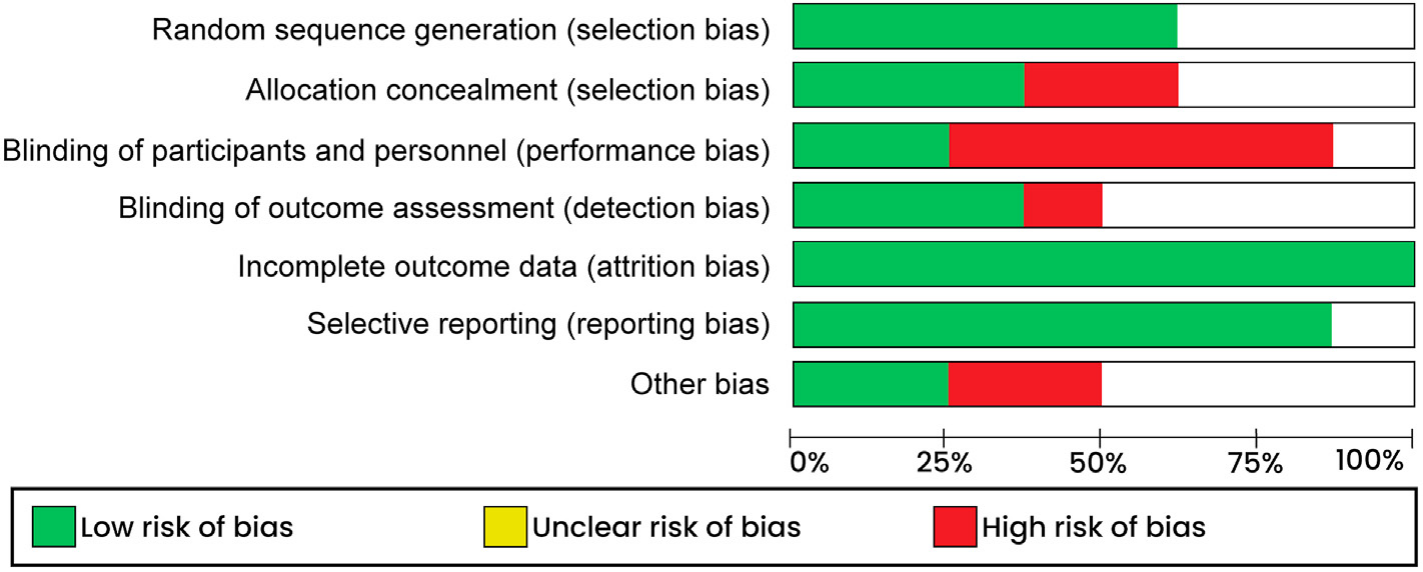
Risk of bias graph.

**Figure 2:**
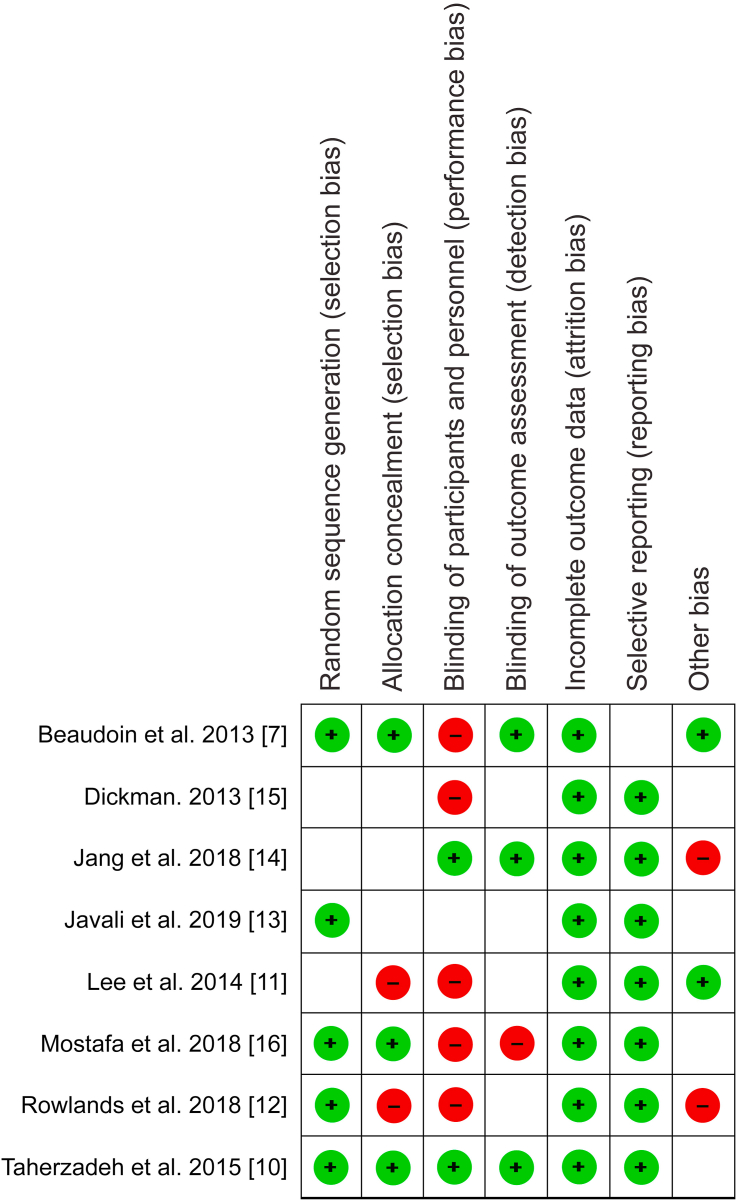
Risk of bias summary.

Quality appraisal of the randomized trials included in present study was independently performed using the risk of bias tool provided in the review manager software (RevMan 5.4.1). The bias in each study element, including selection, attrition, performance and reporting was categorized as either “low risk,” “high risk,” or “unclear risk,” depending on the information provided. Low risk of bias was associated with adequate information, while insufficient information was linked to high risk of bias. Conversely, lack of conclusive judgment on specific elements due to few details resulted in categorization as unclear risk of bias (Figures 1 & 2).

### 2.4 Data extraction

Two reviewers were entrusted with the task of gathering and compiling all pertinent data. The extracted data included; Author ID (Name(s) and year of publishment), characteristics of the included participants, intervention, control intervention, follow-up duration, and main outcomes. Characteristics of participants included Age, sex, and sample size. The intervention section represented the type USGRA used, while the control intervention represented the type of parenteral opioid used and the dosages. All the inconstancies in the retrieved data were reconciled by consulting a third reviewer. The primary outcome in this systematic review was pain scores after USGRA or parenteral opioid application. On the other hand, secondary outcomes were adverse events associated with USGRA and parenteral opioids and the number of patients in need of emergency analgesia.

### 2.5 Data analysis

We carried out a meta-analysis using the Review Manager software (RevMan 5.4.1) to find the pooled effects of USGRA and parenteral opioids. The estimated effect size of continuous outcomes such as pain scores was performed using the mean difference (MD). Conversely, the estimated effect size of binary outcomes such as adverse events was performed using the odds ratio (OR). We also employed a random effect model on all meta-analyses since it takes into account the study heterogeneity. The heterogeneity was assessed through the I^2^ statistics, of which heterogeneity of 25%, 50%, and above 70% was considered low, moderate, and high. The difference in the pooled effect sizes of USGRA and parenteral opioids were deemed statistically significant for p-value of less than 5% (p < 0.05). Forest plots were used to present the meta-analysis of the pooled effect.

## 3 Results

### 3.1 Study selection

**Figure 3:**
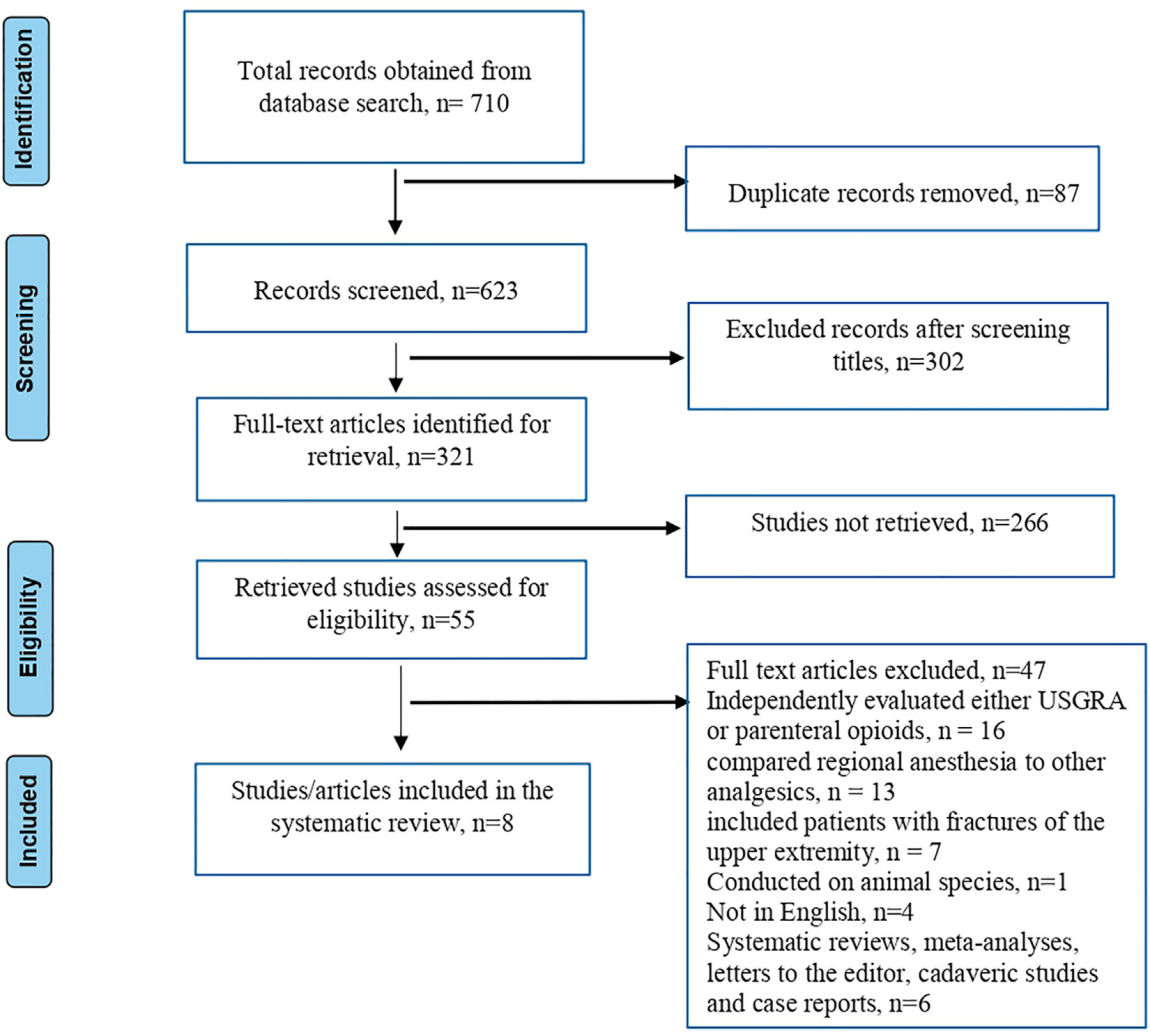
PRISMA flow diagram.

Using the search strategy outlined earlier, 710 articles were identified from the 6 electronic databases. These articles then underwent a duplicate content check, and 87 of them were excluded as they were deemed duplicate articles. An examination of the abstracts and titles of the remaining 623 articles was also carried out resulting in the exclusion of 302 articles. Since we did not retrieve 266 articles, the eligibility criteria were used to assess the remaining 55 articles. Out of these 55 articles, 47 were excluded due to the following reasons; 4 were scientific journals written in other languages, 1 was conducted on animal species, 16 independently evaluated either USGRA or parenteral opioids on patients with hip fractures, 13 compared regional anesthesia to other analgesics, 7 compared USGRA to parenteral opioids on patients with fractures of the upper extremity and 6 were either letters to the editor, systematic review and meta-analyses, cadaveric studies or case reports (see Figure 3). The characteristics of each included study was summarized as shown in Table 1.

**Table 1:** Study characteristics.

Author ID	Participants	Intervention	Control	Main outcomes
Beaudoin et al., 2013 [7]	38 patients (24 female and 14 males aged ≥55 years)	Ultrasound-guided (US) 3-in-1 femoral nerve block (FNB)	Intravenous (IV) morphine	A statistically significant reduction in pain intensity (measured using a numerical rating score (NRS)) was observed in patients enrolled in the FNB group (from 8.3 to 4.3) than in the standard care (SC) group [from 8.0 (5–10) to 8.0 (6–10)] More adverse events were observed in the SC group than FNB group (17 vs. 9) A significantly higher proportion of patients in the FNB group achieved at least a 33% reduction in pain intensity while none of the patients in the SC group achieved a 33% pain reduction.
Taherzadeh et al., 2015 [10]	40 patients (10 female and 30 females aged 5–80 years)	US-guided 3-in-1 FNB	Parenteral morphine sulfate (0.1 mg/kg in adults and 0.05 mg/kg in pediatrics)	Significantly lower pain scores (measured using visual analog scales (VAS)) were observed in the FNB group than morphine group at all intervals (5.20 ± 1.005 vs. 6.70 ± 0.979, 4.30 ± 1.081 vs. 6.55 ± 1.146, 4.50 ± 1.318 vs. 7.20 ± 1.196, and 5.40 ± 1.095 vs. 7.70 ± 1.031, at 15, 30, 60 and 90 min respectively. No complication was observed in the FNB group, while 6 complications were observed in the morphine group.
Lee et al., 2014 [11]	47 patients (33 males and 14 females aged above 65 years)	US-guided 3-in-1 FNB	5 mg of IV morphine for over 2 min, followed by an adjustment to 2.5 mg at 10-minute intervals until the analgesic effect was achieved.	A significant reduction in pain scores was observed in the regional anesthesia group than morphine group (9 of 22 patients in the morphine group and 24 of 25 in the regional anesthesia group achieved a pain reduction to less than 4.) 3 adverse reactions were observed in the morphine group, while 1 patient in the regional anesthesia group experienced an adverse reaction.
Rowlands et al., 2018 [12]	130 patients (28 males and 102 females aged 70 years and above)	US-guided FNB	IV morphine	The cumulative pain scores at rest were significantly higher in the SC group than in the FNB group 4.92 (4.75) vs. 3.16 (3.54) p = 0.043, respectively.) No significant difference was observed in the length of hospital stay (14 (9.25–19.5) 13 (10–18). More patients in the SC group were observed to have delirium; however, when compared to the FNB group, no significant difference was noticed (4/54 (7.4%) vs. 0/54 (0%), p = 0.118). Nausea/vomiting was higher in patients receiving the standard care than intervention group (6/56 (10.7%) vs. 5/51 (9.8%) p = 0.877).
Javali et al., 2019 [13]	60 patients (42 females and 18 males aged >52 years)	US-guided 3-in-1 FNB using Sonosite M-Turbo	IV morphine aimed at achieving a 50% reduction in pain or per-patient request.	Significantly lower NRS scores were observed in the FNB group than SC group (2.0 (1–3) vs. 4.9 (3–7), respectively). More adverse events were observed in the SC group than in the FNB group (23 vs. 12, respectively.) All patients in the FNB group achieved at least 33% pain intensity reduction while only 5 patients in the SC group achieved 33% pain reduction.
Jang et al., 2018 [14]	32 patients (21 female and 11 males aged 61–90 years)	US-guided FNB using a SonoSite S-nerve instrument with a 6 MHz linear array transducer	IV tramadol aimed at achieving a 50% reduction in pain or per-patient request.	A significantly lower VAS score was observed in the FNB than in the Standard management (SM) group (3.62 vs. 7.06, p < 0.001 and 4.5 vs. 5.75, p < 0.001, at 4 and 24 h, respectively). More adverse events were observed in the SM group than FNB group (13 vs. 9, respectively). Significantly less rescue IV tramadol was required by patients in the FNB group than SM group (12.5 ± 9.12 vs. 53.7 ± 37.7 mg, p = 0.001, respectively.)
Dickman., 2013 [15]	64 patients (23 males and 41 females aged 18–75 years)	US-guided FNB and US-guided Fascia iliaca compartment block (UFIB)	IV morphine administered at 0.1 mg/kg	Patients in the US-guided FNB group showed a significant reduction in pain at 30 min to patients in the UFNB group and IV morphine group (1.94 (2.43) vs. 2.05 (2.61) vs. 5.13 (2.73) p < 0.0001, respectively) 60 min after analgesia administration UFIB group showed significantly lower pain scores than FNB and IV morphine groups (1.90 (2.38) vs. 2.58 (3.06) vs. 4.40 (2.90), p < 0.05, respectively). At 480 min, no significant difference in pain scores between the UFNB, UFIB, and IV morphine groups was observed (3.20 (2.28) vs. 2.35 (3.07) vs. 3.74 (2.89) p = 0.342, respectively.) No adverse event was reported in either group.
Mostafa et al., 2018 [16]	60 patients (42 males and 18 females)	Patient-controlled US-guided fascia iliaca compartment analgesia (PC-FICA)	Patient-controlled IV fentanyl (PC-IVF) administered at 20 μg/ml	Statistically lower VAS scores were observed in the PC-FICA group than in the PC-IVF group at 1h, 3h, and 6h postoperatively (p < 0.05). Significantly lower number of patients in the PC-FICA group required rescue analgesia than in the PC-IVF group (7 vs. 19, p = 0.03) No significant difference in patient satisfaction rates was observed in either group (24 (80%) vs. 27 (90%) for PC-IVF and PC-FICA groups, respectively.) More complications were observed in the PC-IVF than in the PC-FICA group (6 vs. 3, respectively).

### 3.2 Primary outcome

**Figure 4:**
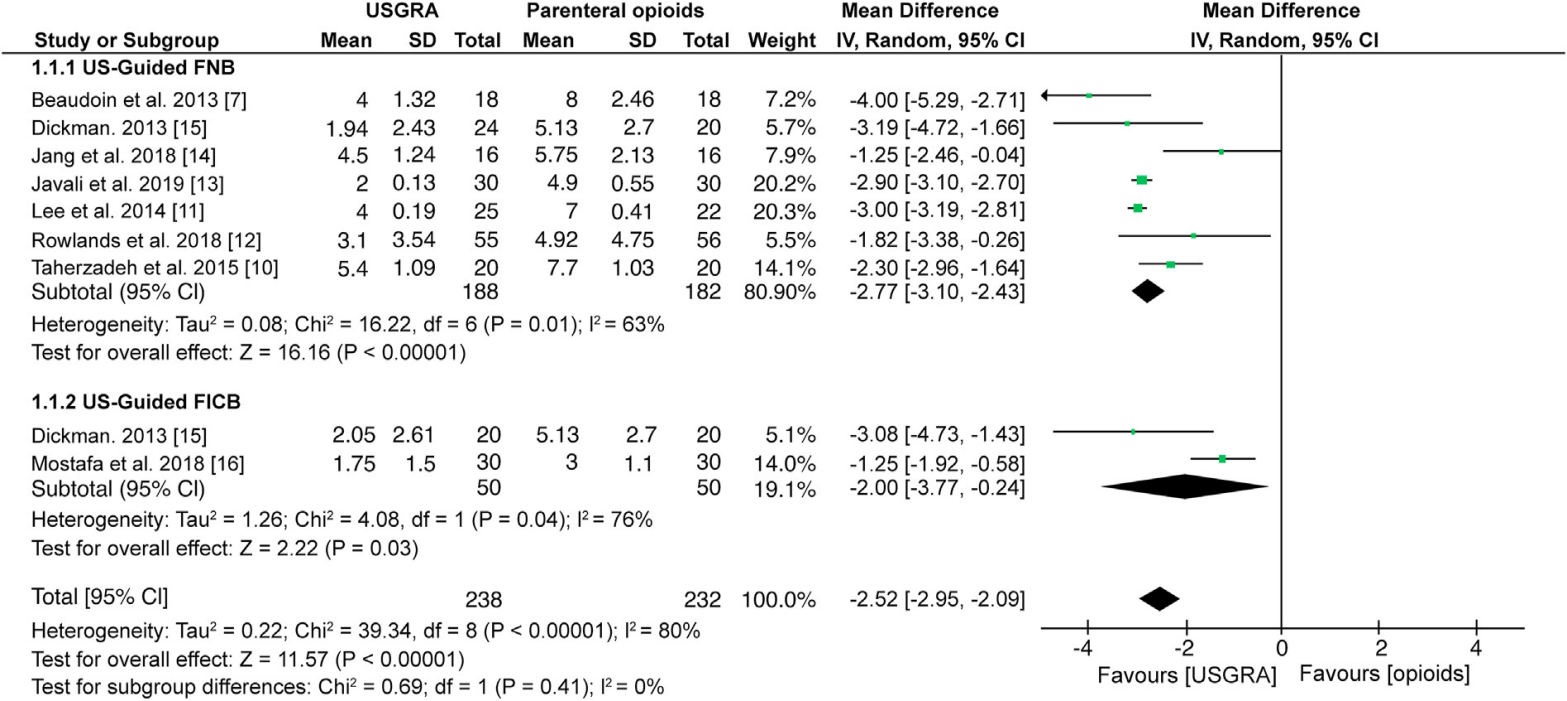
Forest plot showing a comparison between USGRA and parenteral opioids on pain intensity.

The main outcome of this systematic review was the evaluation of pain reduction, which was reported in all the studies. The results of a subgroup meta-analysis including 370 patients shows that US-guided FNB significantly reduced the pain intensity than parental opioids (MD; -2.77: 95% CI; -3.10, -2.43; p < 0.00001; I^2^ = 63%). Similarly, a subgroup meta-analysis of data from 100 patients shows that US-guided FICB significantly reduced pain intensity than parenteral opioids (MD; -2.00: 95% CI; -3.77, -0.24; p = 0.03; I^2^ = 76%) (Figure 4).

### 3.3 Secondary outcomes

**Figure 5:**
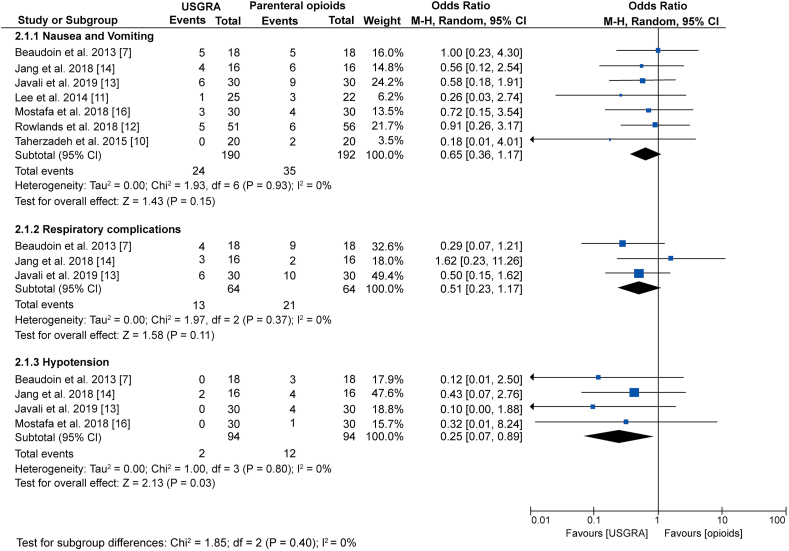
Forest plot showing comparison between USGRA and parenteral opioids on the occurrence of adverse events.

**Figure 6:**
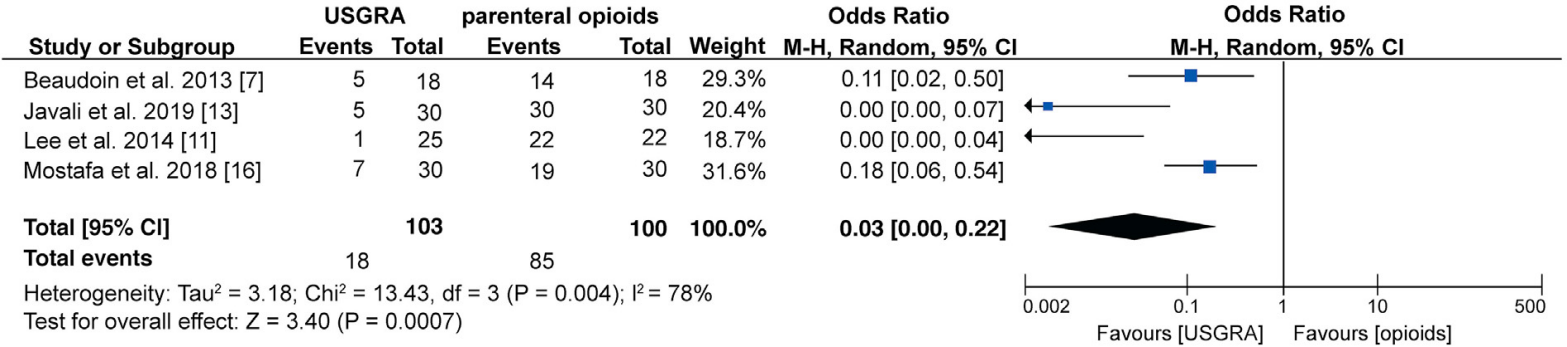
Forest plot showing comparison between USGRA and parenteral opioids on the need for rescue analgesia.

The main adverse reactions related to USGRA and parenteral opioids were nausea, vomiting, respiratory complications, and hypotension reported in 7 studies that included 382 patients. A subgroup analysis of data from 7 studies showed no statistically significant difference in the occurrence of nausea and vomiting between the two groups (OR; 0.65: 95% CI; 0.36, 1.17; p = 0.15; I^2^ = 0%). A subgroup analysis of data from 3 studies also showed no significant difference on the incidence of respiratory complications between the two groups (OR; 0.51: 95% CI; 0.23, 1.17; p = 0.11; I^2^ = 0%). However, a subgroup analysis of data from 4 studies has shown a significant difference in the incidence of hypotension between the two groups, with USGRA showing a significantly lower rate (OR; 0.25: 95% CI; 0.07, 0.89; p = 0.03; I^2^ = 0%) (Figure 5). Similarly, a meta-analysis of data from 4 studies has shown that significantly fewer patients in the USGRA group required rescue analgesia than the patients in the parenteral opioids group (OR; 0.03: 95% CI; 0.00, 0.22; p = 0.0007; I^2^ = 78%) (Figure 6).

## 4 Discussion

The primary goal of the current study was to compare the efficacy of USGRA to parenteral opioids for analgesia in hip fracture patients. The results of our meta-analysis show that USGRA had a significantly better overall pain reduction than parenteral opioids. A subgroup meta-analysis also showed significantly lower hypotension events in the USGRA group than in the parenteral opioids group. However, a subgroup analysis showed no significant difference in respiratory complications and events of vomiting or nausea in either group. Our meta-analysis also revealed that the need for rescue analgesia was significantly reduced by using USGRA for analgesia than parenteral opioids.

The results of our meta-analysis have supported our initial hypothesis that USGRA would significantly reduce pain scores than parenteral opioids. These results are consistent with the results presented by a recent meta-analysis that compared USGRA to other control analgesic treatments, including parenteral opioids [17]. The meta-analysis showed that USGRA significantly reduced pain after block placement than the conventional therapy (MD; -2.35: 95% CI; -3.07, 1.62: p < 0.00001). Similarly, another meta-analysis evaluating ultrasound-guided Serratus Anterior Plane Block (SAPB) Combined with General anesthesia reported that the pain scores were significantly lower in the SAPB group than in the control group. The pooled results from this study showed that at 6 h, 12 h and 24 h the VAS pain scores were significantly lower in the SAPB group. An earlier study also suggested that for musculoskeletal pain, regional anesthesia be used as an alternative or adjunct to intravenous opioids [18]. However, research indicates that the utilization of regional anesthesia in the emergency ED is restricted. An Australian questionnaire survey of multiple emergency departments revealed that out of 646 hip fractures, regional anesthesia was used in only 45 fractures [19]. Similarly, a recent nationwide UK survey showed that regional anesthesia was used in only 44% of emergency departments for analgesia in patients with hip fractures [20]. According to this study, the main reasons for the low use of regional anesthesia were a shortage of skilled personnel and equipment.

The most commonly used regional analgesia in hip fracture patients are fascia iliaca compartment block and femoral nerve block. Previous studies have shown that these methods were performed blindly using the anatomical landmarks in the emergency department [6]. However, using anatomical landmarks to administer regional anesthesia carries the risk of undesirable outcomes such as nerve damage and vascular perforation, and preparing nerve stimulants to avoid side effects is difficult [11]. Therefore, using ultrasound guidance in employing regional analgesia helps to perform the analgesia more rapidly and safely, which makes it more suitable for use in the emergency department. Our meta-analysis has shown that employing US-Guided FNB and FICB for analgesia significantly reduced the pain scores in patients with hip fractures. This systematic review did not compare the two methods as it was not the essence of the study; however, previous studies have provided the comparison. For example, a study by Yu et al. [21] evaluated the use of US-guided continuous FNB to US-guided continuous FICB for analgesia in 60 elderly patients with hip fractures. Results of this study showed that 6 h after surgery FICB group had a significantly lower mean VAS at rest than the FNB group (0.5 ± 0.8 vs. 1.0 ± 1.3, respectively). However, a 2020 observational study revealed that at 5 min, both blocks significantly reduced pain [22]. Results of this study show that the FNB group had significantly lower VAS (visual analog score) pain scores than the FICB group (2.1 ± 1.4 vs. 3.3 ± 1.1, p < 0.05, respectively). Another study which included 100 patients with fractures of the neck of the femur demonstrated that US-Guided FNB was not superior to US-Guided FICB [23]. This study showed significant reductions in the mean pain scores for patients that were subjected to FICB and FNB (2.62 and 2.3, for FICB and FNB); however, when the pain scores were compared, there was no significant difference (p = 0.408).

The use of USGRA and parenteral opioids is also associated with several complications. We hypothesized that USGRA would have significantly fewer adverse events than parenteral opioids. The results show that only hypotension was significantly lower in USGRA than in parenteral opioids. This significant difference in hypotension can be associated with the fact that in some studies, the intravenous opioids were administered in repeated doses to achieve at least 50% reduction in pain. Other adverse reactions such as vomiting, nausea, and respiratory complications showed no significant difference. Similarly, a recent meta-analysis comparing US-guided SAPB to conventional analgesics showed no significant difference in the adverse events between the SAPB and control groups [24]. However, another meta-analysis comparing US-guided peripheral nerve blocks (US-PNB) to conventional analgesia reported that the risk of serious adverse events was significantly lower in the US-PNB (MD = 25.91; p < 0.001; 95% CI; 19.74 to 32.07; I^2^ = 76%) [17]. Delirium events have also been reported in some trials. For instance, Rowlands et al. [12] after comparing FNB to IV morphine among 141 patients with neck of femur fractures revealed that the presence of delirium was observed in 4 patients. In contrast, none of the patients in the FNB group had delirium. Similarly, a 2019 study reported that US-Guided FICB reduces postoperative delirium in patients with hip fractures [25]. Results of this study revealed that a significantly lower incidence of postoperative delirium was observed in the experimental group than in the control group (6 (13.9%) vs. 15 (35.7%), p = 0.018, respectively). Similar results were reported in a meta-analysis that compared US-PNB to control analgesics including parenteral opioids. The meta-analysis of 4 trials revealed that US-PNB significantly reduces the incidence of delirium than control (RR 0.60, p = 0.03; 95% CI 0.38 to 0.94; I^2^ = 0%) [17]. However, other studies have reported an insignificant difference in the presence of delirium between USGRA and control analgesics. Morrison et al. [26] compared the use of US-FNB to standard analgesics, including both oral and intravenous opioids, in 161 patients with hip fractures and found no significant difference in the incidence of delirium in either group (17.1% vs. 15.9%, p = 0.83, for control and intervention, respectively). Other adverse reactions such as pruritus and bradycardia have also been reported in the included studies. Mostafa et al. [16] reported that 1 patient in the patient controlled intravenous fentanyl (PC-IVF) group developed bradycardia while none of the patient in the patient controlled fascia iliaca compartment analgesia (PC-FICA) group developed any event of bradycardia. On the other hand, Jang et al. [14] reported only a single case of pruritus which was observed in the standard management group.

Our meta-analysis also shows that USGRA is associated with significant reduction in the need for rescue analgesia (OR; 0.03: 95% CI; 0.00, 0.22; p = 0.0007; I^2^ = 78%). The studies have shown that different types of rescue analgesia and varied dosages have been used. Beaudoin et al. [7] reported that parenteral morphine was used as the rescue analgesia for all patients requiring rescue analgesia. The study also reports that hydromorphone was used as rescue analgesia in 3 patients while fentanyl was used in only one patient. The rescue opioid dosage used in this trial ranged from 2 to 6 mg and 2–21 mg for patients in the FNB and Standard care (SC) groups, respectively. Similarly, Lee et al. [11] reported that all patients in the IV morphine required additional morphine injections. The results show that rescue morphine consumption was significantly higher in the IV morphine group patients than in the regional anesthesia group (11.4 ± 4.9 vs. 0.4 ± 2.0 mg, respectively). A 2019 study also showed that morphine was the rescue analgesic of choice [13]. The results revealed that significantly lower morphine consumption was observed in the FNB group than SC group (0.8 (0–6) vs. 9.5 (7–12), p < 0.001, respectively). Additionally, Mostafa et al. [16] reported that the rescue analgesic used was fentanyl. Patients in the PC-IVF group showed significantly higher consumption of rescue analgesia than PC-FICA (70.5 ± 20.4 vs. 31.4 ± 10.7, p < 0.05, respectively).

Even though our meta-analysis has shown no significant difference in most of the adverse events, evidence from some of the included studies has shown that USGRA is associated with increased patient satisfaction than parenteral opioids. Mostafa et al. reported that more patients in the PC-FICA group were satisfied than patients in the PC-IVF group; however, the difference was insignificant. Previous studies comparing USGRA to other analgesic methods also show that USGRA is associated with increased patient satisfaction. A recent study comparing US-Guided FICB to a control group receiving either paracetamol, tramadol, or morphine depending on the VAS scores reported that a statistically significant increase in patient satisfaction was observed in the FICB group than the control group (23.6 vs. 17.9; P = 0.01) [27]. Ma et al. [28] also compared US-Guided continuous FICB with a control group receiving peroral tramadol and paracetamol and found that patient satisfaction was significantly higher in the study group than in the control group (45.68 ± 11.29 vs. 74.77 ± 9.52 p < 0.001).

Hip fractures are associated with pain, which determines the length of hospital stay. It is reported that adequate analgesia is associated with a reduction in pain among hip fracture patients, which in turn reduces the length of hospital stay [29, 30]. Only two studies in this systematic evaluated the length of hospital stay among patients with hip fractures. Rowlands et al. [12] showed that the length of hospital stay for patients in the intervention group was lower than in the SC group; however, the difference was statistically insignificant (13 (10–18) vs.14 (9.25–19.5) p = 0.89, respectively). Lee et al. [11] also reported no significant difference in the length of hospital stay between the IV morphine and regional anesthesia groups (355.8 ± 174.4 vs. 343.0 ± 149.4 mins, p = 0.787, respectively). The results of these trials are consistent with a recent meta-analysis showing no significant difference in length of hospital stay between US-PNB and control group (MD − 0.92 days; p = 0.49; 95% CI − 3.55 to 1.71; I 2 = 86%). During the hospital stay or follow-up, period mortality may also be witnessed among patients receiving either USGRA or parenteral opioids. Rowlands et al. [12] reported that 2 days after analgesia administration, one patient in the Intervention group died. Similarly, a previous study by Hao et al. [25] reported one case of mortality which was witnessed in the FICB group. The study reports that this death occurred 4 h postoperatively due to pulmonary embolism.

### 4.1 Limitations

The primary limitation of this systematic review is that the number of trials comparing USGRA to parenteral opioids alone were limited; thus, the analyzed results may be biased and should be interpreted with caution. The other limitation is the high heterogeneity observed in the meta-analyses comparing pain reductions and the need for rescue analgesia. The high heterogeneity in pain reduction can be attributed to the fact that different pain score measurements were used. In some studies, pain scores were measured using VAS, while others were measured using numerical rating score (NRS). Despite the high heterogeneity, the results of our meta-analyses were not affected as it established that USGRA was superior to parenteral opioids in the reduction of pain and need for rescue analgesia. The dosages of parenteral opioids used in the studies were also different, which may have had a different effect on the pain relief. The inclusion criteria of this systematic review also allowed for the inclusion of studies published in English only. This may have led to the omission of some vital information that may have otherwise enhanced the results of our meta-analysis.

## 5 Conclusion

The results of our study have shown that both US-guided FNB and US-guided FICB were superior to parenteral opioids in the management of hip fracture pain. Similarly, the results showed that USGRA decreased the need for rescue analgesia compared to parenteral opioids. The number of adverse effects observed in the USGRA group was lower than those observed in the parenteral opioids; however, the difference was not statistically significant. The only significant difference in adverse effects was observed in the reduction of hypotension. This significant difference was attributed to the fact that in some of the studies, the parenteral opioids were administered in repeated doses to achieve at least 50% reduction in pain. Future studies should also be designed to examine the effect of USGRA compared with parenteral opioids on other additional outcomes such as delirium, length of hospital stay, and mortality rates. Future studies should also compare the long-term effects of USGRA and parenteral opioids on pain relief. The superiority of USGRA in pain relief and the need for rescue analgesia shows that it is an effective and safe alternative to parenteral opioids. Therefore, based on the results of our analysis, we can advocate the use of USGRA in analgesia for hip fracture patients. Future systematic reviews comparing the different techniques of USGRA in analgesia for patients with hip fractures should also be performed to understand the effective technique.

### 5.1 Article summary

1) Why is this topic important?

Hip fracture is a common presentation in the emergency department. Emergency physicians play a significant role in providing safe and effective pain control measures for hip fracture patients which can be challenging in some instances.

2) What does this review attempt to show?

USGRA significantly lower the pain intensity and have significantly fewer adverse events than parenteral opioids.

3) What are the key findings?

USGRA is superior to parenteral opioids in reducing pain scores and the need for rescue analgesia.

4) How is patient care impacted?

The superiority of USGRA in pain relief and the need for rescue analgesia shows that it is an effective and safe alternative to parenteral opioids. Therefore, based on the results of our analysis, we can advocate the use of USGRA in analgesia for hip fracture patients.

## Declarations

### Author contribution statement

All authors listed have significantly contributed to the development and the writing of this article.

### Funding statement

This research did not receive any specific grant from funding agencies in the public, commercial, or not-for-profit sectors.

### Data availability statement

Data included in article/supp. material/referenced in article.

### Declaration of interest’s statement

The authors declare no competing interests.

### Additional information

No additional information is available for this paper.

## References

[1] Chami G., Jeys L., Freudmann M., Connor L., Siddiqi M. (2006). Are osteoporotic fractures being adequately investigated?: a questionnaire of GP & orthopaedic surgeons. BMC Fam. Pract..

[2] Braithwaite R.S., Col N.F., Wong J.B. (2003). Estimating hip fracture morbidity, mortality and costs. J. Am. Geriatr. Soc..

[3] Schnell S., Friedman S.M., Mendelson D.A., Bingham K.W., Kates S.L. (2010). The 1-year mortality of patients treated in a hip fracture program for elders. Geriatr. Orthop. Surg. Rehabil..

[4] Penrod J.D., Litke A., Hawkes W.G. (2008). The association of race, gender, and comorbidity with mortality and function after hip fracture. J. Gerontol.: Series A.

[5] Jones J.S., Johnson K., McNinch M. (1996). Age as a risk factor for inadequate emergency department analgesia. Am. J. Emerg. Med..

[6] Fletcher A.K., Rigby A.S., Heyes F.L.P. (2003). Three-in-one femoral nerve block as analgesia for fractured neck of femur in the emergency department: a randomized, controlled trial. Ann. Emerg. Med..

[7] Beaudoin F.L., Haran J.P., Liebmann O. (2013). A comparison of ultrasound-guided three-in-one femoral nerve block versus parenteral opioids alone for analgesia in emergency department patients with hip fractures: a randomized controlled trial. Acad. Emerg. Med..

[8] Marhofer P., Schrögendorfer K., Wallner T., Koinig H., Mayer N., Kapral S. (1998). Ultrasonographic guidance reduces the amount of local anesthetic for 3-in-1 blocks. Reg. Anesth. Pain Med..

[9] Marhofer P., Schrögendorfer K., Koinig H., Kapral S., Weinstabl C., Mayer N. (1997). Ultrasonographic guidance improves sensory block and onset time of three-in-one blocks. Anesth. Analg..

[10] Taherzadeh D., Jahanian F., Montazer H., Bozorgi F., Aminiahidashti H., Hosseininejad M., Golikhatir I. (2015).

[11] Lee H.K., Kang B.S., Kim C.S., Choi H.J. (2014). Ultrasound-guided regional anesthesia for the pain management of elderly patients with hip fractures in the emergency department. Clin. Exp. Emerg. Med..

[12] Rowlands M., Walt van de G., Bradley J. (2018). Femoral nerve block intervention in neck of femur fracture (FINOF): a randomised controlled trial. BMJ Open.

[13] Babu Homanna Javali R., Gb S., A P., Srinivasarangan M., S J., N S., Sb A. (2019). Efficacy of ultrasound-guided 3-in-1 femoral nerve block for pain management in elderly patients presenting to the emergency department with hip fractures: a randomized controlled trial. IJAA.

[14] Jang J.S., Lee Y.-H., Kandahar H.K. (2018). Changes in the tumor necrosis factor-α level after an ultrasound-guided femoral nerve block in elderly patients with a hip fracture. Braz. J. Anesthesiol.(English Edition).

[15] Dickman E. (2013). https://clinicaltrials.gov/ct2/show/results/NCT01904071.

[16] Mostafa S.F., Eid G.M., Elkalla R.S. (2018). Patient-controlled fascia iliaca compartment block versus fentanyl patient-controlled intravenous analgesia in patients undergoing femur fracture surgery. Egypt. J. Anaesth..

[17] Exsteen O.W., Svendsen C.N., Rothe C., Lange K.H.W., Lundstrøm L.H. (2022). Ultrasound-guided peripheral nerve blocks for preoperative pain management in hip fractures: a systematic review. BMC Anesthesiol..

[18] De Buck F., Devroe S., Missant C., Van de Velde M. (2012). Regional anesthesia outside the operating room: indications and techniques. Curr. Opin. Anaesthesiol..

[19] A H., Sa S., S H. (2010). Patterns of analgesia for fractured neck of femur in Australian emergency departments. Emerg. Med. Australasia (EMA) : Emerg. Med. Australasia (EMA).

[20] Rashid A., Beswick E., Galitzine S., Fitton L. (2014). Regional analgesia in the emergency department for hip fractures: survey of current UK practice and its impact on services in a teaching hospital. Emerg. Med. J..

[21] Yu B., He M., Cai G.-Y., Zou T.-X., Zhang N. (2016). Ultrasound-guided continuous femoral nerve block vs continuous fascia iliaca compartment block for hip replacement in the elderly. Medicine (Baltim.).

[22] Gupta M., Kamath S.S. (2020). Comparison of preoperative ultrasound guided fascia iliaca block versus femoral nerve block for proximal femur fractures before positioning for spinal anesthesia: an observational study. Kor. J. Pain.

[23] Cooper A.L., Nagree Y., Goudie A., Watson P.R., Arendts G. (2019). Ultrasound-guided femoral nerve blocks are not superior to ultrasound-guided fascia iliaca blocks for fractured neck of femur. Emerg. Med. Australasia (EMA).

[24] Zhang X., Zhang C., Zhou X., Chen W., Li J., Wang H., Liu J. (2020). Analgesic effectiveness of perioperative ultrasound-guided Serratus anterior Plane block combined with general anesthesia in patients undergoing video-assisted thoracoscopic surgery: a systematic review and meta-analysis. Pain Med..

[25] Hao J., Dong B., Zhang J., Luo Z. (2019). Pre-emptive analgesia with continuous fascia iliaca compartment block reduces postoperative delirium in elderly patients with hip fracture. A randomized controlled trial. Saudi Med. J..

[26] Morrison R.S., Dickman E., Hwang U. (2016). Regional nerve blocks improve pain and functional outcomes in hip fracture: a randomized controlled trial. J. Am. Geriatr. Soc..

[27] Thompson J., Long M., Rogers E., Pesso R., Galos D., Dengenis R.C., Ruotolo C. (2020). Fascia iliaca block decreases hip fracture postoperative opioid consumption: a prospective randomized controlled trial. J. Orthop. Trauma.

[28] Ma Y., Wu J., Xue J., Lan F., Wang T. (2018). Ultrasound-guided continuous fascia iliaca compartment block for pre-operative pain control in very elderly patients with hip fracture: a randomized controlled trial. Exp. Ther. Med..

[29] Chin R.P.-H., Ho C.-H., Cheung L.P.-C. (2013). Scheduled analgesic regimen improves rehabilitation after hip fracture surgery. Clin. Orthop. Relat. Res..

[30] Dulaney-Cripe E., Hadaway S., Bauman R., Trame C., Smith C., Sillaman B., Laughlin R. (2012). A continuous infusion fascia iliaca compartment block in hip fracture patients: a pilot study. J. Clin. Med. Res..

